# Monitoring interdecadal coastal change along dissipative beaches via satellite imagery at regional scale – Corrigendum

**DOI:** 10.1017/cft.2024.3

**Published:** 2024-02-16

**Authors:** Marcan Graffin, Mohsen Taherkhani, Meredith Leung, Sean Vitousek, George Kaminsky, Peter Ruggiero

**Affiliations:** 1 LEGOS (CNES/CNRS/IRD/UT3), Université de Toulouse, Toulouse, France; 2 Lab’OT (CNES), Toulouse, France; 3 Department of Civil and Construction Engineering, Oregon State University, Corvallis, OR, USA; 4 College of Earth, Ocean, and Atmospheric Sciences, Oregon State University, Corvallis, OR, USA; 5 Pacific Coastal and Marine Science Center, U.S. Geological Survey, Santa Cruz, CA, USA; 6 Department of Civil, Materials, and Environmental Engineering, University of Illinois Chicago, Chicago, IL, USA; 7 Washington State Department of Ecology, Olympia, WA, USA

When this article was originally published in Coastal Futures it contained an error in some dates displayed in its abstract. This has been updated along with some minor syntactical changes in the text.

The authors apologise for these errors.

## References

[r1] Graffin M, Taherkhani M, Leung M, Vitousek S, Kaminsky G and Ruggiero P. (2023)Monitoring interdecadal coastal change along dissipative beaches via satellite imagery at regional scale. Cambridge Prisms: Coastal Futures 1, e42. 10.1017/cft.2023.30 PMC12337611 40881949

