# Supplementary material for: Monitoring interdecadal coastal change along dissipative beaches via satellite imagery at regional scale
Source: Camb Prism Coast Futur. 2023 Dec 4;1:e42. doi: 10.1017/cft.2023.30 (PMC12337611; doi:10.1017/cft.2023.30)
Supplement: Graffin et al. supplementary material [file S2754720523000306sup001.pdf]

Monitoring interdecadal coastal change along dissipative beaches via satellite imagery at regional scale

Marcan Graffin[1,2,*], Mohsen Taherkhani[3], Meredith Leung[4], Sean Vitousek[5,6], George Kaminsky[7] and Peter Ruggiero[4]

[1] Université de Toulouse, LEGOS (CNES/CNRS/IRD/UT3), Toulouse, France
[2] Lab'OT (CNES), Toulouse, France
[3] Department of Civil and Construction Engineering, Oregon State University, Corvallis, OR, USA
[4] College of Earth, Ocean, and Atmospheric Sciences, Oregon State University, Corvallis, OR, USA
[5] Pacific Coastal and Marine Science Center, U.S. Geological Survey, Santa Cruz, CA, USA
[6] Department of Civil, Materials, and Environmental Engineering, University of Illinois Chicago, Chicago, IL, USA
[7] Washington State Department of Ecology, Olympia, WA, USA
* Correspondence: Marcan Graffin (Email: marcan.graffin@ird.fr)

USGS pre-decisional disclaimer: This draft manuscript is distributed solely for purposes of scientific peer review. Its content is deliberative and pre-decisional, so it must not be disclosed or released by reviewers. Because the manuscript has not yet been approved for publication by the U.S. Geological Survey (USGS), it does not represent any official USGS finding or policy.

Author Contributions

M.G. and P.R. developed the initial concept for this study. M.G., M.T., and M.L. processed the data. M.G. performed and verified the analysis. M.G. and M.T. wrote the original manuscript. All authors discussed the results and edited the manuscript.

Competing Interests

The authors declare no competing interests.

Data Availability

The satellite-derived MHW shoreline data set generated and analysed in this study is available in the following Zenodo data repository : `https://zenodo.org/uploads/10136946`.
Beach elevation profile data from the in-situ monitoring program can be found on the following website (NANOOS-NVS): `https://nvs.nanoos.org/BeachMapping`.

Supplementary

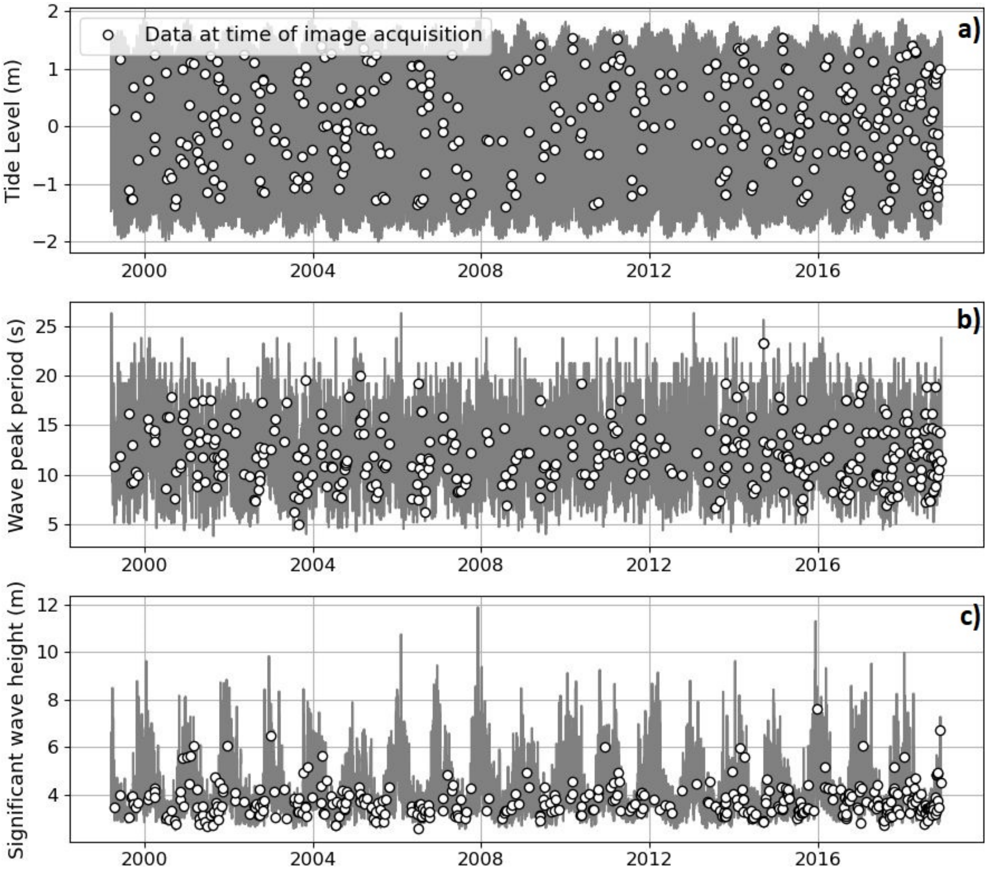

Figure S1: Time series of **(a)** measured tide level (NOAA tide gauge ID 9439040 [datum is MSL] [1]), **(b)** peak wave period, and **(c)** significant wave height (both obtained from the CAWCR/CSIRO wave hindcast product [2]) at Astoria, Oregon, during 1999-2019. The white dots correspond to the time of the image acquisition on a site (transect) along the Long Beach subcell, Washington.

S	n	$\overline{R^2}$	$\overline{RMSE}$ (m)	$\overline{bias}$ (m)
10	1	0.52	22.76	7.69
15	1	0.51	21.58	7.16
10	2	0.41	31.21	5.99
15	2	0.42	29.45	7.25

Table S1: $\overline{R^2}$, $\overline{RMSE}$, and $\overline{bias}$ validation scores obtained for the tide-correction of the SDW time series (correction table 4 in Table 1) for different pairs of *hampel* function input parameters, S and n. The methodology used for obtaining these scores is described in Section 3.

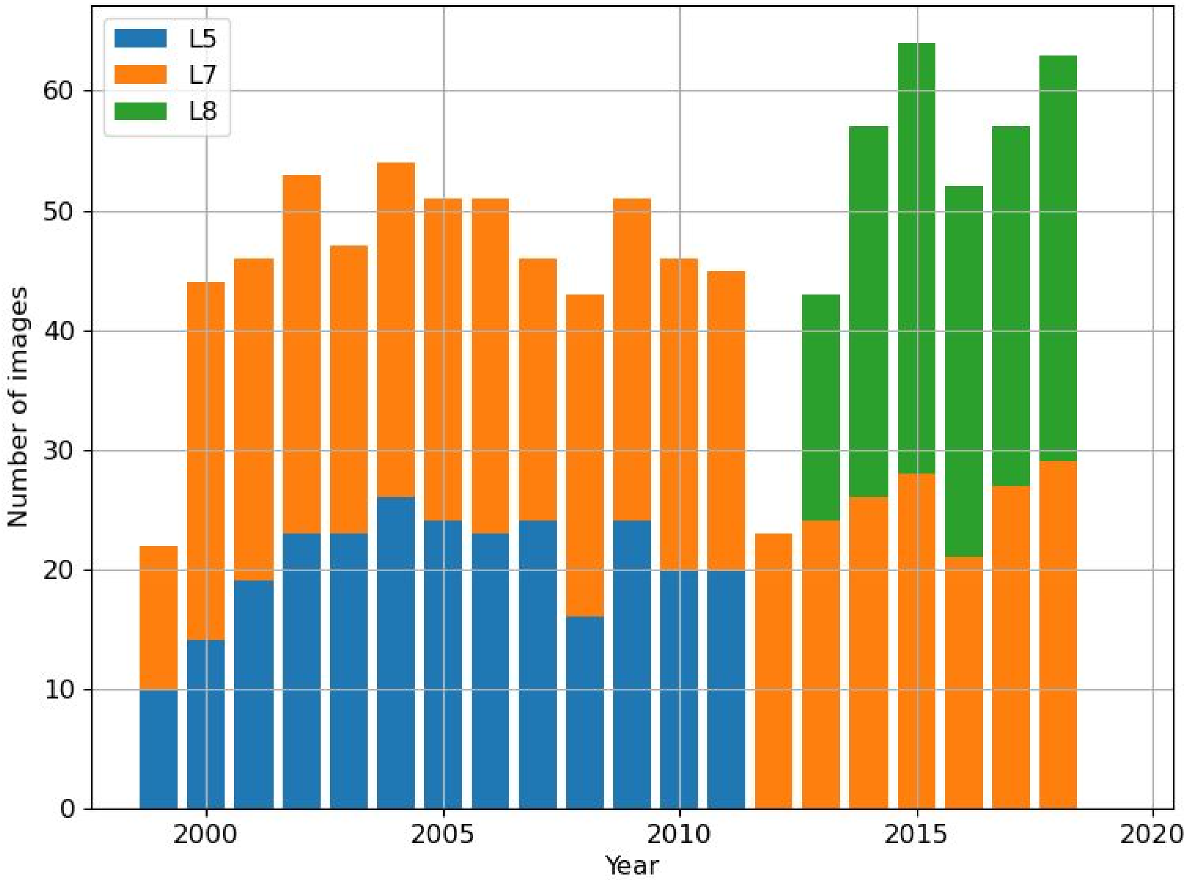

Figure S2: Temporal evolution of the average across all transects number of images used per each Landsat satellite mission.

References

[1] NOAA, "Astoria, OR - Station ID: 9439040." `https://tidesandcurrents.noaa.gov/stationhome.html?id=9439040`.

[2] T. Durrant, M. Hemer, G. Smith, C. Trenham, and D. Greenslade, "Cawcr wave hindcast - aggregated collection. v5.." `http://hdl.handle.net/102.100.100/137152?index=1`, 2019.

[3] K. Vos, K. Splinter, M. Harley, J. Simmons, and I. Turner, "Coastsat: A google earth engine-enabled python toolkit to extract shorelines from publicly available satellite imagery," *Environmental Modelling & Software*, 2019.

[4] M. O. Pedrido, "hampel filter." `https://github.com/MichaelisTrofficus/hampel_filter`, 2021.

[5] P. Ruggiero, G. Kaminsky, G. Gelfenbaum, and B. Voigt, "Seasonal to interannual morphodynamics along a high-energy dissipative littoral cell," *Journal of Coastal Research*, 2005.

[6] WDE, "Beach and shoreline change." `https://nvs.nanoos.org/BeachMapping`.

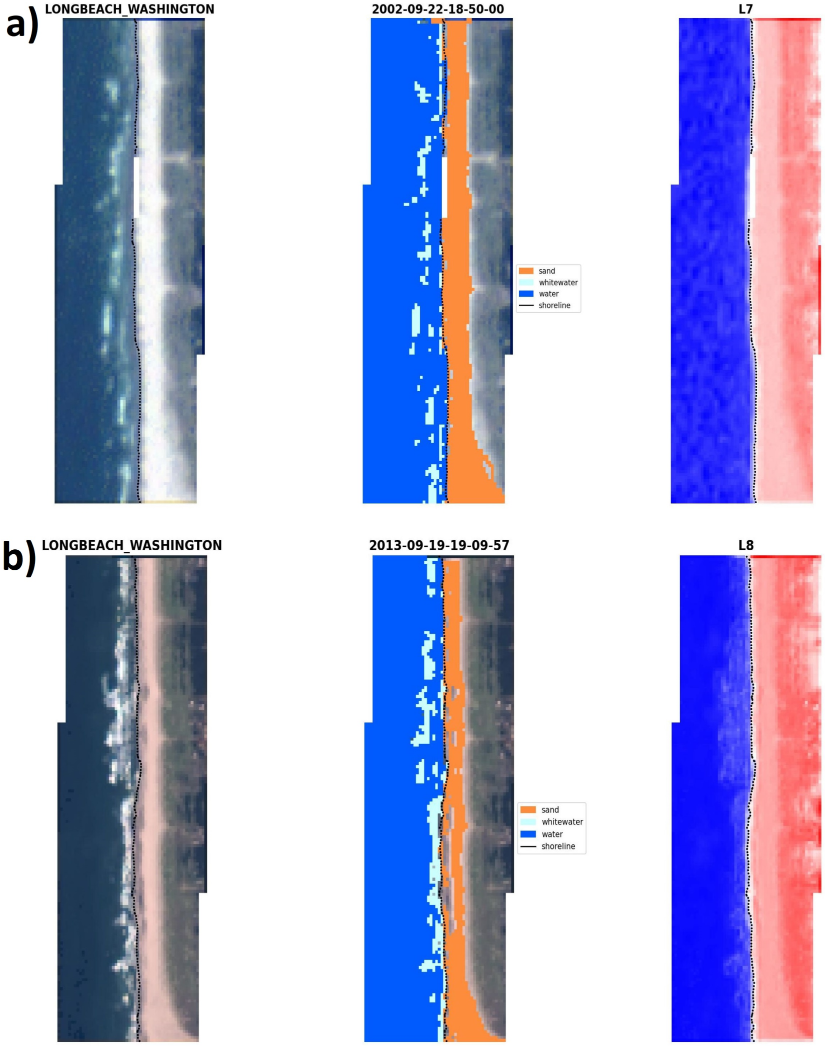

Figure S3: Examples (in Long Beach sub-cell, Washington) of good cases of **(a)** Landsat 7 and **(b)** Landsat 8 images. The left-side, middle, and right-side panels respectively represent the RGB, pixel-classified, and MNDWI-classified images. Figure obtained using CoastSat (*Vos et al., 2019* [3])

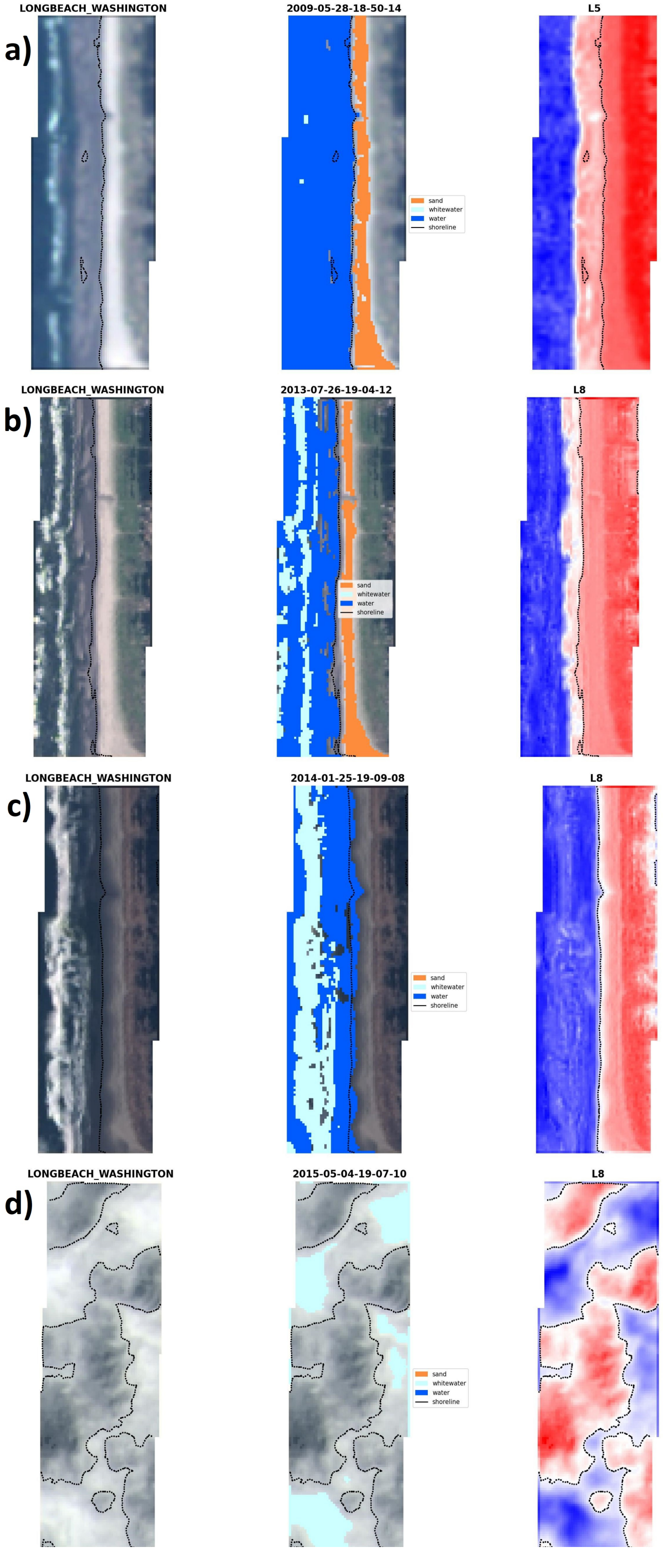

Figure S4: Examples (in Long Beach sub-cell, Washington) of misdetection of the waterline in different cases. Panel (a) shows a misdetection during a low tide, where the detected waterline is confounded with the wet/dry sand interface, and patches of shoreline are detected on the wet sand. Panel (b) depicts a misdetection during a low tide, where the detected waterline is roughly close to the wet/dry sand interface. Panel (c) demonstrates a misdetection during a high-energy wave event, where the classification of the sand pixels failed, and the detected waterline stands between the wet/dry sand interface and the waterline. Lastly, panel (d) represents a misdetection due to the presence of unmasked clouds.Figure obtained using CoastSat (*Vos et al., 2019* [3])

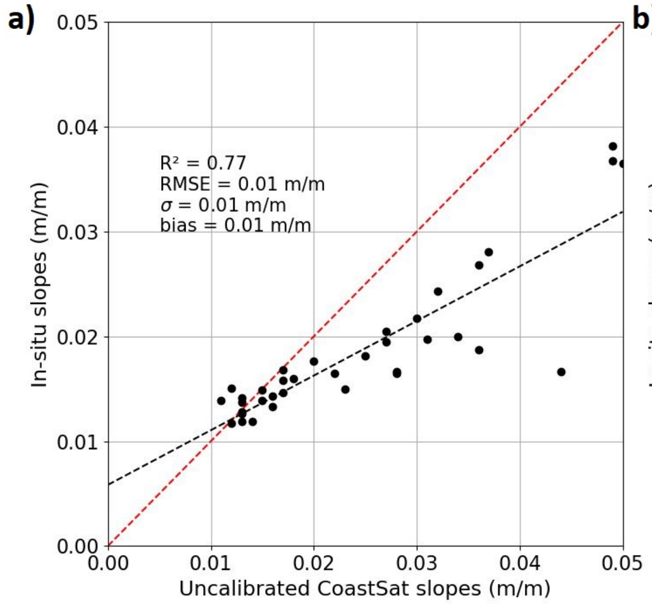

Figure S5: Direct comparison of in-situ beach slopes obtained between MSL and MHW elevations over the 42 validations sites versus **(a)** the raw (uncalibrated) CoastSat-derived beach slopes and **(b)** the calibrated CoastSat-derived beach slopes. Uncalibrated slopes are obtained using the CoastSat-Slopes tool (*Vos et al., 2019* [3])

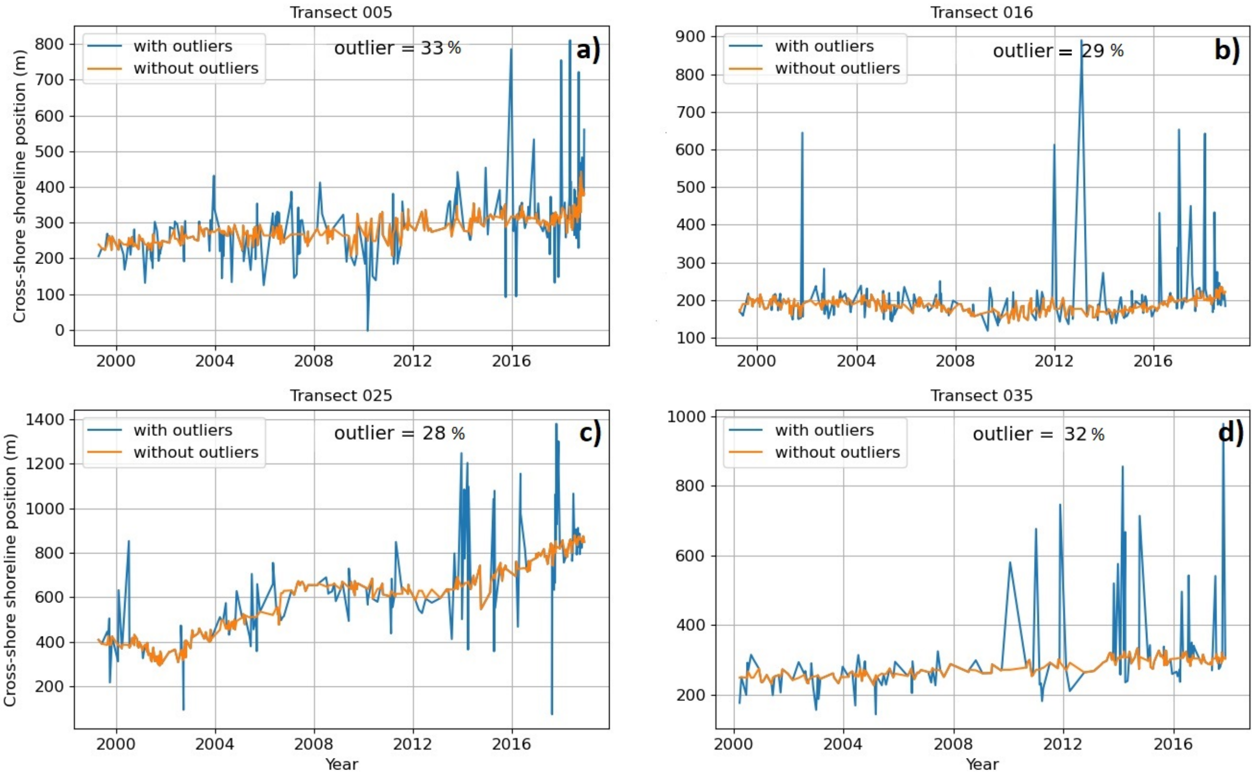

Figure S6: Four examples of time series of cross-shore shoreline position extracted via CoastSat (*Vos et al., 2019* [3]). The blue and orange lines show the raw and the outlier-corrected SDS time series [4], respectively (both after the tide and wave corrections have been applied)

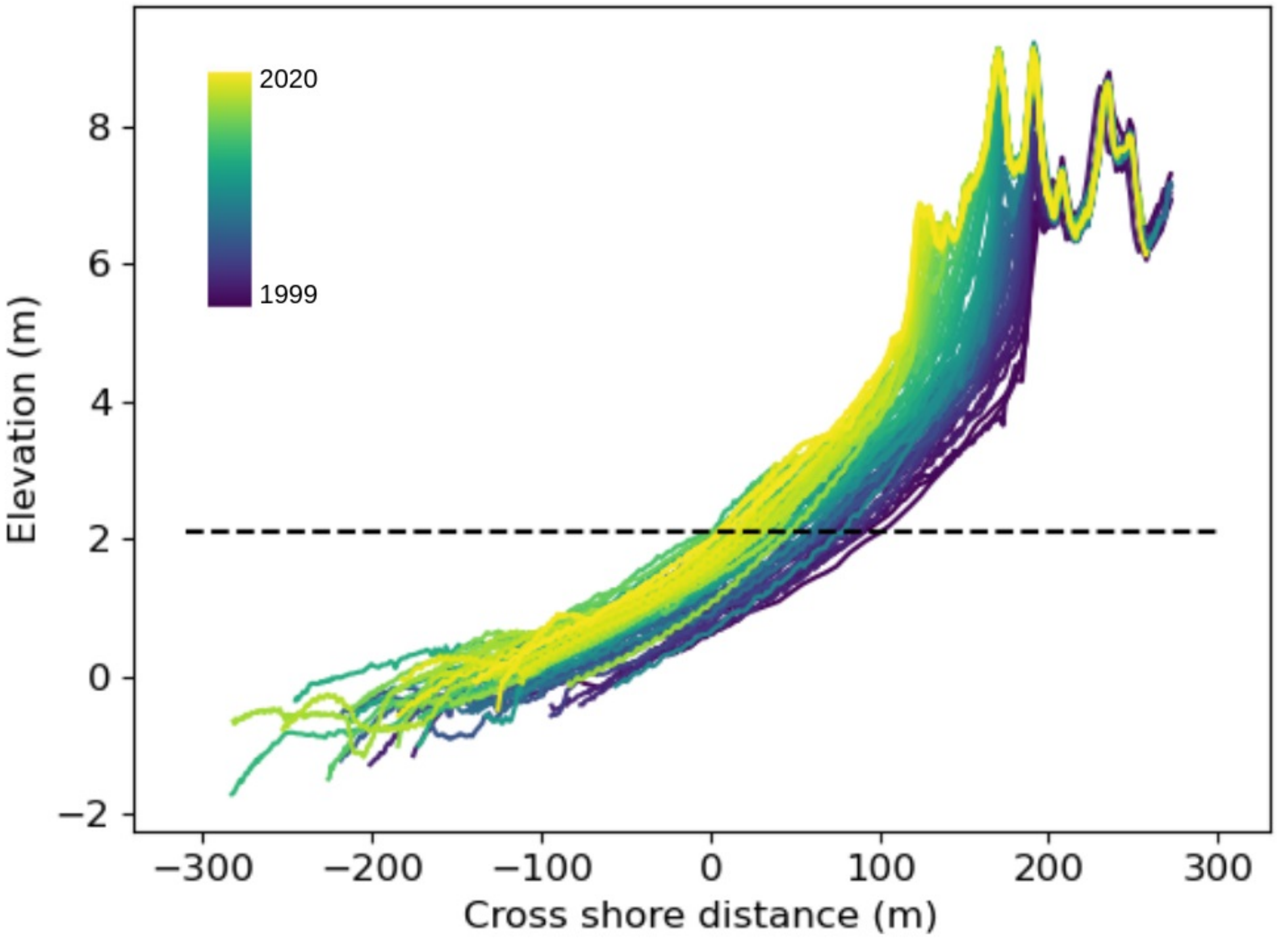

Figure S7: Elevation profiles measured every ∼3 months during 1999-2020 for a site (profile 020) in the Long Beach subcell, Washington (shown in Fig. 1a). The older profiles are shown in dark blue shades, and the more recent ones are in yellow. The dashed black line shows the MHW level, i.e., the 2.1 m elevation (NAVD88). Data obtained as part of the measurement program initiated by *Ruggiero et al. (2005)* [5], these data are made publicly available by the *Washington State Department of Ecology* [6].

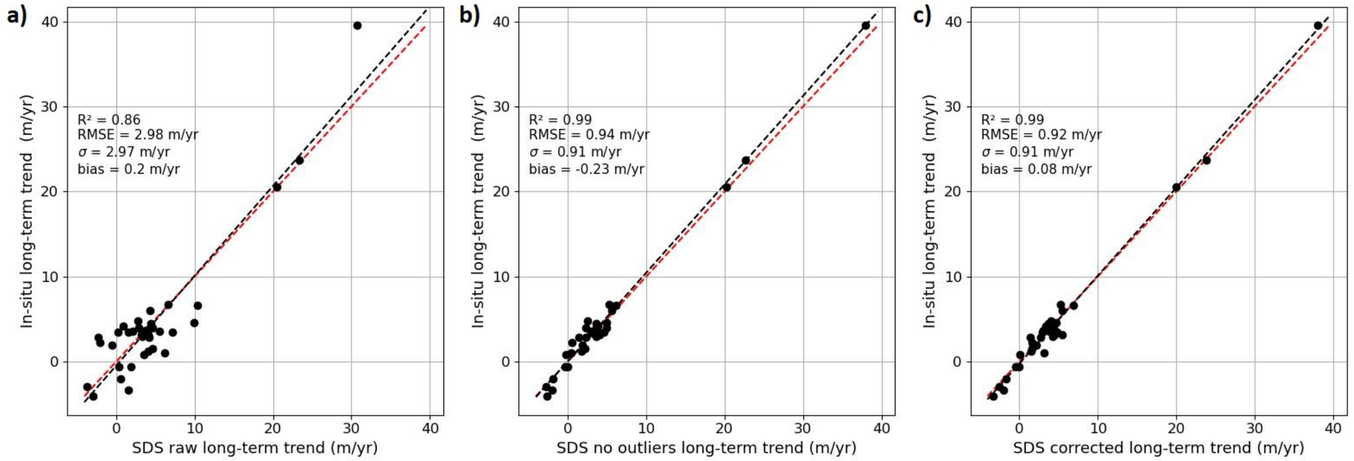

Figure S8: Direct comparison of the shoreline change trends (1999-2018) over the 42 validation sites from the satellite-derived and in-situ shoreline positions for **(a)** raw SDS data, **(b)** outlier-corrected SDS data, and **(c)** outlier- and tide-corrected SDS data.

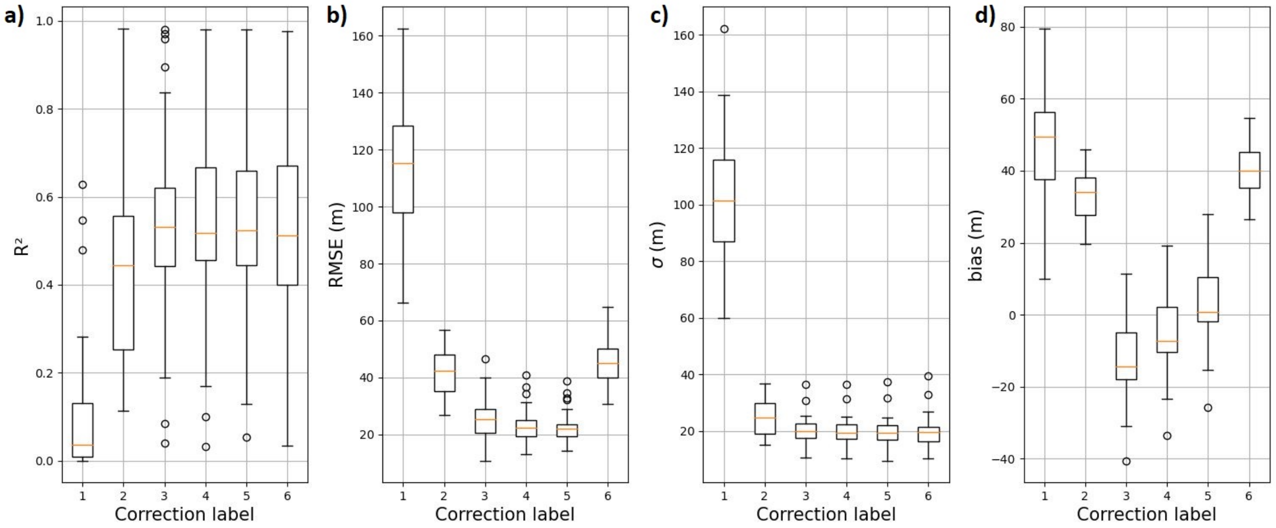

Figure S9: Similar to Fig. 4, but the corrections are made using the time-averaged (static) beach slopes computed from the beach profiles for each transect (from elevations between MSL and MHW).

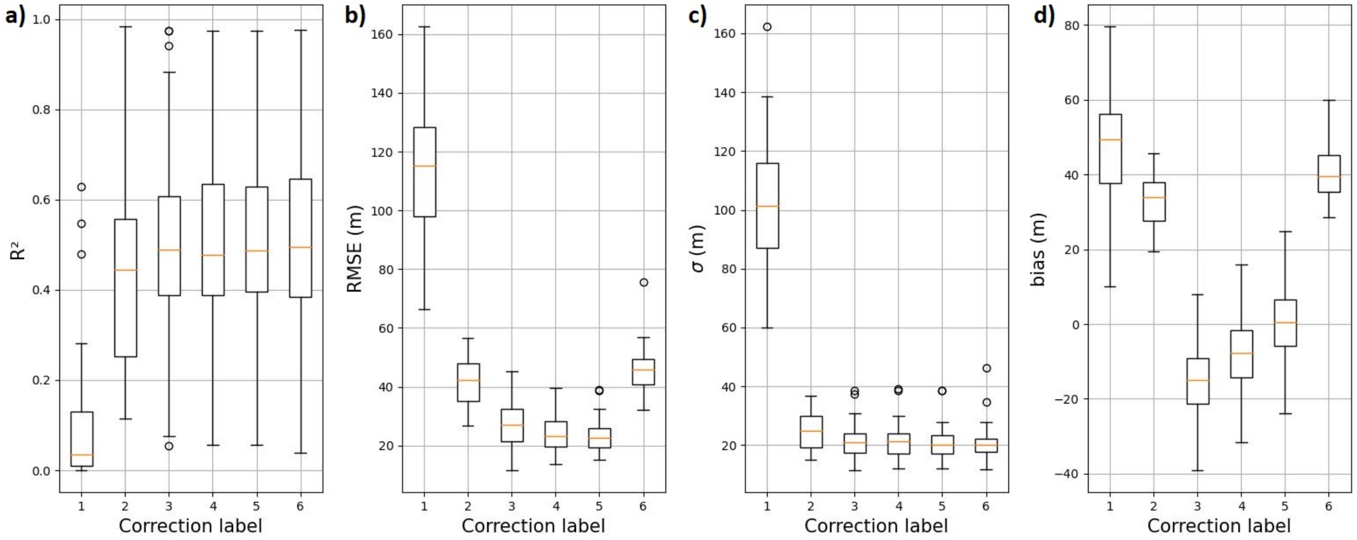

Figure S10: Similar to Fig. 4, but the corrections are made using the time-dependent (dynamic) beach slopes computed from the beach profiles for each transect (from elevations between MSL and MHW).
